# Profiling ADC targets in cholangiocarcinoma: implications for therapeutic development

**DOI:** 10.1038/s41698-025-01139-6

**Published:** 2025-11-19

**Authors:** Mari Nakazawa, Waqar Arif, Ezra Baraban, Andres Matoso, Elsa Hallab, Emma Kartalia, Nilofer S. Azad, Marina Baretti, Daniel J. Zabransky, Robert A. Anders, Kiyoko Oshima, Mark Yarchoan

**Affiliations:** 1 Department of Oncology, Johns Hopkins University School of Medicine, Baltimore, MD USA; 2 Department of Pathology, Johns Hopkins University School of Medicine, Baltimore, MD USA

**Keywords:** Biomarkers, Cancer, Oncology

## Abstract

Antibody drug conjugates (ADCs) are clinically active in several cancers, but their relevance in cholangiocarcinoma (CCA) is undefined. We profiled 23 CCA tumors and found that TROP2 was highly expressed (82.6%) but also present in benign epithelium. NECTIN4 (65.2%) showed tumor-specific staining, supporting selectivity. B7-H3 (47.8%) and CLDN18.2 (13.0%) were less frequently positive. IDH1-mutant tumors demonstrated attenuated ADC target expression. These data provide rationale for evaluating ADC strategies in CCA.

## Introduction

Antibody-drug conjugates (ADCs) are a rapidly expanding class of oncology drugs that have improved outcomes for patients with a variety of solid tumor malignancies^1–6^. By coupling cytotoxic agents to antibodies targeting tumor-associated antigens, ADCs may enhance tumor specificity and reduce the systemic toxicity typical of traditional chemotherapy. Advances in antigen selection and linker–payload chemistry have broadened their clinical utility, including in malignancies with limited treatment options^7^.

Despite recent progress, cholangiocarcinoma (CCA) remains a disease with limited therapeutic options and a median survival of only approximately one year in the advanced/metastatic setting. Although traditionally considered a rare cancer, the incidence of CCA is rising^8^, paralleling trends in obesity and other etiologic risk factors. In the U.S., an estimated 10,000 new cases are diagnosed annually, with most patients presenting with advanced or unresectable disease^9^. First-line therapy for CCA consists of gemcitabine and cisplatin with immune checkpoint inhibitors^10,11^, but response rates remain modest and most patients without targetable mutations ultimately receive FOLFOX in the second-line setting, a regimen associated with a overall response rate of 5%^12^. There is thus an urgent need to identify novel therapeutic targets in CCA.

Approximately 10% of CCA express HER2, with higher rates observed in extrahepatic compared to intrahepatic tumors^13^, for which there are data supporting the use of the HER2-targeting ADC trastuzumab deruxtecan (T-DXd), now approved in a tumor-agnostic setting^14^. However, the expression of other clinically relevant ADC targets in CCA remains largely unexplored. To address this gap, we profiled the expression of four ADC targets in CCA: NECTIN4, TROP2, CLDN18.2, and B7-H3. These targets are known to be expressed in other solid tumors and are the focus of FDA-approved or investigational ADCs, but their expression in CCA is not well characterized.

### Clinical cohort

Our final clinical cohort included 23 patients (Table 1) with primarily intrahepatic (91.3%) CCA with a median age of 60 (range 33–88) at time of surgery. A majority of the patients were white (82.6%) and female (52.2%). Most patients represented in this cohort (65.2%) had clinically actionable mutations, most commonly *FGFR2* fusions (34.8%) and *IDH1* mutations (21.7%). Most surgical specimens were from primary liver (82.6%) or bile duct (4.3%) resections, while the remaining represented sites of metastasis (12.9%). Over half of patients (52.2%) represented received systemic therapy prior to surgery.

**Table 1:** Baseline demographic and tumor characteristics in patients with cholangiocarcinoma (CCA, *n* = 23) who underwent primary tumor or metastatic site resection

	Total Cohort (*n* = 23)
Age at surgery	
Mean (SD)	59.4 (12.8)
Median [Min, Max]	60.0 [33.0, 80.0]
Gender	
Female	12 (52.2%)
Male	11 (47.8%)
Race	
Black	4 (17.4%)
White	19 (82.6%)
Pre-surgery CA19-9	
CA19-9 Abnormal	10 (43.5%)
CA19-9 Normal	11 (47.8%)
Missing	2 (8.7%)
Specimen site	
Common Bile Duct	1 (4.3%)
Femur	1 (4.3%)
Jejunum	1 (4.3%)
Liver	19 (82.6%)
Pancreas	1 (4.3%)
Subtype	
Extrahepatic	1 (4.3%)
Hilar	1 (4.3%)
Intrahepatic	21 (91.3%)
Receipt of systemic therapy	
Yes	12 (52.2%)
No	11 (47.8%)
Mutation status	
FGFR2	8 (34.8%)
IDH1	5 (21.7%)
BRCA2	1 (4.3%)
HER2	1 (4.3%)
None	2 (8.7%)
Other	4 (17.4%)
Not done	2 (8.7%)
NECTIN4 H-score	
Mean (SD)	82.4 (112)
Median [Min, Max]	10.0 [0, 300]
TROP2 H-score	
Mean (SD)	121 (99.2)
Median [Min, Max]	100 [0, 300]
CLDN18.2 H-score	
Mean (SD)	10.0 (33.2)
Median [Min, Max]	0 [0, 150]
B7H3 H-score	
Mean (SD)	28.8 (50.2)
Median [Min, Max]	0 [0, 180]

### NECTIN4 and TROP2 are commonly expressed in cholangiocarcinoma

**Fig. 1:**
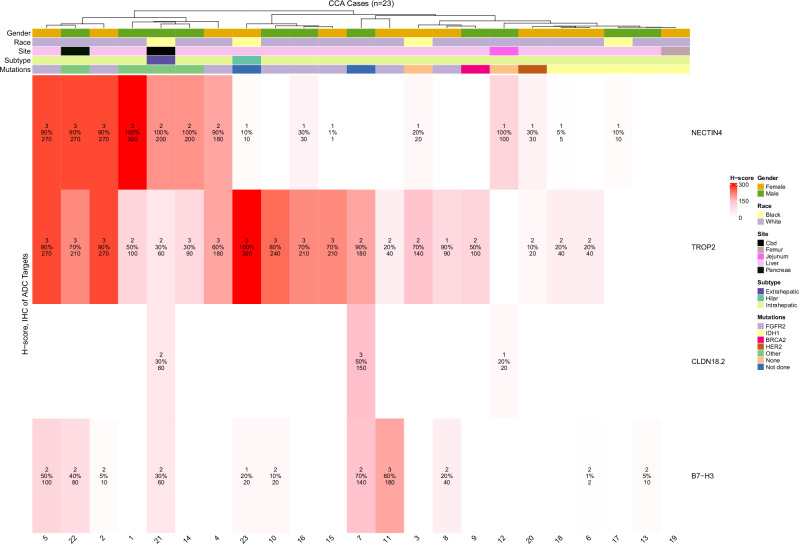
ADC target expression in cholangiocarcinoma (CCA) tumor specimens. Heatmap of NECTIN4, TROP2, CLDN18.2, and B7-H3 expression across 23 cholangiocarcinoma cases from institutional archival specimens. Each column represents an individual patient. The color gradient reflects H-score, with white indicating no expression and deep red indicating high expression (H score range 0–300). Annotations within each heatmap cell indicate intensity (first value), percentage positivity (second value), and resultant H-score (third value). Tumor and clinical characteristics are shown in the top annotation bars, including gender, race, tumor site, subtype (intrahepatic, hilar, and extrahepatic), and presence of actionable mutations (*FGFR2*, *IDH1*, *BRCA2*, *HER2*, other, none, or testing not performed).

**Fig. 2:**
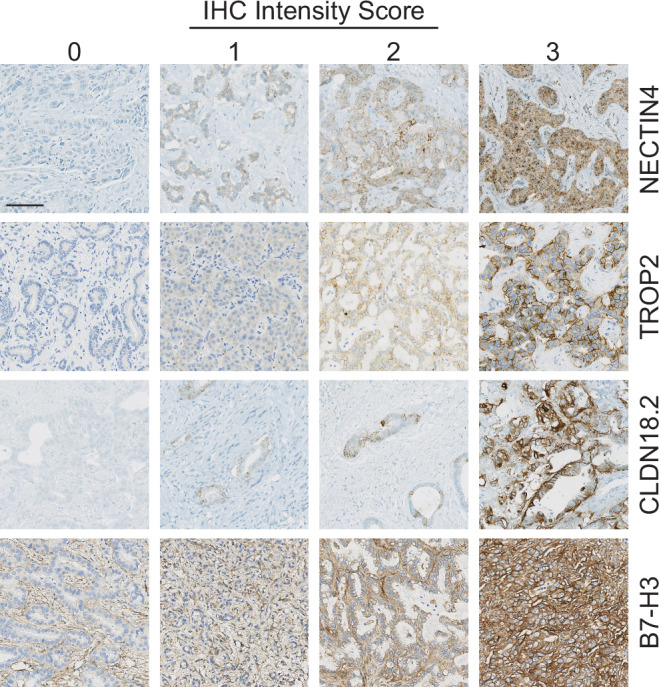
Representative immunohistochemistry (IHC) staining intensities for ADC targets in cholangiocarcinoma. Representative images of tumor tissue sections stained for NECTIN4, TROP2, CLDN18.2, and B7-H3, illustrating the spectrum of IHC intensity scores (0 = negative, 1 = weak, 2 = moderate, 3 = strong).

Of the four ADC targets surveyed (Figs. 1 and 2), TROP2 expression was most commonly seen across all specimens, in 82.6% of patients (3+ in 39.1%; H score range 20–300). NECTIN4 positivity was observed in 65.2% of patients (3+ in 17.4%; H score range 1–300), followed by B7-H3 positivity in 47.8% (3+ in 4.3%; H-score range 2–180), and CLDN18.2 positivity in 13.0% (3+ in 4.3%; H score range 20–150). NECTIN4 and CLDN18.2 expression were highly specific to malignant bile ducts, with no background staining observed. In contrast, TROP2 expression was also detected in benign biliary epithelium, at times with higher intensity than in malignant tissue (Supplementary Fig. 1). Similarly, B7-H3 staining lacked tumor specificity and was observed in adjacent tumor-associated stroma and cancer-associated fibroblasts (CAFs).

### IDH1 mutation status is associated with low ADC target expression

Next, we sought to understand whether global ADC expression patterns differed by concominant mutation status (Supplementary Fig. 2). *IDH1* mutated tumors were significantly associated with lower composite ADC expression compared with *IDH1* wildtype tumors (*p* < 0.001). *FGFR2* rearranged tumors were associated with numerically increased target expression, though not statistically significant (*p* = 0.13).

This study provides initial evidence that several ADC targets are variably expressed in CCA, with implications for therapeutic development. NECTIN4 and TROP2 were frequently expressed, at times at high intensity, supporting their potential relevance as ADC targets in this disease.

Despite accounting for both intensity and extent of staining, the H-score may overestimate biological relevance in cases of diffuse low-intensity expression, which are likely less meaningful for ADC efficacy than focal high-intensity staining. Clinical experience with T-DXd has shown that tumors with strong IHC positivity exhibit higher objective response rates compared to those with low-intensity expression^15^, underscoring the importance of ADC target expression intensity as a predictive biomarker. In addition to target expression, effective payload delivery is shaped by the tumor microenvironment (TME). The dense desmoplastic stroma characteristic of CCA can restrict intratumoral drug distribution, and stromal barriers have been linked to reduced penetration of other cancer therapies^16^. Such features may also contribute to the relatively modest response rates reported with T-DXd in biliary tract cancers compared with other tumor types^17^. Strategies to modulate the stroma and improve drug delivery may therefore represent an important avenue for future investigation.

NECTIN4, targeted by the FDA-approved ADC enfortumab vedotin^2^, was expressed with moderate to high intensity (2+ or 3+) in 30.4% of cases, and was restricted to tumor cells, making it a compelling target. In contrast, CLDN18.2 and B7-H3 were less commonly expressed, suggesting that these targets may have more limited utility in unselected CCA populations. TROP2 expression at 3+ intensity was observed in 39.1% of cases; however, staining was also seen in benign biliary epithelium, at times exceeding that of the adjacent tumor. This raises considerations for off-tumor payload delivery, particularly in the setting of biliary inflammation or epithelial remodeling, which may be unique to the pathophysiology of CCA. While biliary toxicity has not been reported in phase III trials of the TROP2-targeting ADC sacituzumab govitecan^4,6^, benign expression in target tissues underscores the need for careful safety monitoring in future ADC studies in this population.

Although limited by small numbers, *IDH1* mutated tumors were significantly associated with reduced ADC expression, a finding that may reflect the hypermethylation phenotype^18^, as well as broader epigenetic reprogramming or a progenitor-like cell of origin with distinct transcriptional programs^19,20^ characteristic of this subset. In contrast, *FGFR2* rearranged tumors showed comparable ADC expression to wild-type cases, particularly when considering that the five low-expressing *IDH1* mutant tumors fell within the *FGFR2* wild-type subgroup. These exploratory findings suggest potential molecular correlates of ADC target expression in CCA.

This analysis is limited by the small sample size and retrospective nature of the cohort, which may constrain the generalizability of our findings. Our cohort had an overrepresentation of patients with intrahepatic cholangiocarcinoma (91.3%) and those with targetable mutations (65.2%), which may not fully reflect the broader CCA population. Furthermore, though there are previous studies reporting on TROP2 expression as a negative prognostic marker^21^, we were not able to analyze correlations between ADC target expression and clinical outcomes, as the specimens spanned more than a decade during which substantial changes in standard-of-care therapies occurred. These temporal shifts introduce confounding that limits the interpretability of outcome data in a small, heterogeneous cohort. There remain unanswered questions regarding other features of ADC expression in CCA, including the relationship between the expression at primary tissue versus metastatic site. Nevertheless, our findings highlight potential feasibility of ADC-based strategies in CCA. Future validation in larger, contemporary cohorts, utilizing tissue microarrays from primary and metastatic derived from patients with clinical outcomes data, will be essential to confirm these findings and help prioritize ADC targets for prospective clinical investigation.

## Methods

### Patient cohort

We retrospectively identified surgical specimens from patients with histologically confirmed CCA who underwent resection at Johns Hopkins Hospital between 2015 and 2024. Both intrahepatic and extrahepatic CCA cases were included. Clinical data, including patient demographics and tumor characteristics, including concomitant mutation status by standard of care NGS, were abstracted from the electronic medical record. All patients represented in this study provided written informed consent prior to participation.

### Quantitative immunohistochemistry

Immunohistochemistry (IHC) was performed on 5 μm-thick formalin-fixed, paraffin-embedded (FFPE) tissue sections evaluate expression of four ADC targets: NECTIN4, TROP2, CLDN18.2, and B7-H3. Staining was conducted in a CLIA-certified laboratory. Detailed information on antibody sources, concentrations, and staining platforms is provided in Supplementary Table 1. IHC expression was qualitatively scored based on staining intensity using a 3-point scale: 1 (weak), 2 (moderate), and 3 (strong, readily visible at low magnification). Only cytoplasmic and/or membranous staining was considered positive. H-scores were calculated by multiplying the percentage of positive lesional cells by the staining intensity (e.g., 70% of cells with moderate (2+) staining = H-score of 140). Staining intensity was assessed independently by two pathologists blinded to clinical outcomes, with scores recorded separately for each target antigen; discrepancies were resolved by joint review and consensus.

### Analytical and statistical methods

Analytical and statistical methods were primarily descriptive. Heatmaps were constructed to illustrate IHC positivity patterns for the four ADC targets, with column annotations for race, gender, CCA subtype, site of specimen, and concomitant mutation status. For analysis of ADC positivity trends by concomitant mutational status, composite ADC positivity was calculated as the mean of H-scores across all four targets for each specimen. Box plots were generated to compare composite ADC expression by mutational status, with *IDH1* mutant and *FGFR2* rearranged tumors analyzed relative to wild-type cases using the Wilcoxon rank-sum test. Analysis was performed using RStudio software v4.4.2 (2024-10-31).

## Supplementary information

Supplemental Materials

## Data Availability

The datasets generated and/or analysed during the current study are not publicly available due to the inclusion of patient data, but are available from the corresponding author on reasonable request.

## Code availability

All analyses were performed using R with standard packages for data cleaning, visualization, and statistical analysis. No custom algorithms were developed.
