# Supplementary material for: Profiling ADC targets in cholangiocarcinoma: implications for therapeutic development
Source: NPJ Precis Oncol. 2025 Nov 19;9:364. doi: 10.1038/s41698-025-01139-6 (PMC12630836; doi:10.1038/s41698-025-01139-6)
Supplement: Supplementary file 1 — Supplemental Materials [file 41698_2025_1139_MOESM1_ESM.docx]

**Figure S1:** Representative image of TROP2 staining of tumor (3 X 30 = H-score 90) and adjacent benign parenchyma (bile duct 3 x 100 = H-score 300).

**Figure S2: Composite ADC expression by *IDH1* mutation and *FGFR2* rearrangement statuses.** Boxplots depict composite ADC expression (mean H-score across NECTIN4, TROP2, CLDN18.2, and B7-H3) stratified by mutation status. Statistical comparisons were performed using two-sided Wilcoxon rank-sum tests.

**Supplemental Table 1:** Antibodies used for staining of ADC targets (NECTIN4, TROP2, CLDN18.2, B7-H3)

| **Antibody** | **Source** | **Catalog #** | **Species** | **Concentration** | **Staining Platform** | **Detection** |
| --- | --- | --- | --- | --- | --- | --- |
| CLDN18.2 | Roche | 790-7027 | Mouse | 3 µg/mL | Ventana | UltraView DAB |
| NECTIN4 | abcam | ab192033 | Rabbit | 13 µg/mL | Roche | Optiview |
| TROP2 | Santa Cruz | sc-376181 | Mouse | 5 µg/mL | Leica | Bond Refine Dab |
| B7-H3 | Cell Signaling Technology | 14058S | Rabbit | 0.1 µg/mL | Leica | Bond Refine Dab |
